# Serologic Evidence of Hantavirus Exposure in Humans, Italy, 2004–2018

**DOI:** 10.3201/eid3209.260840

**Published:** 2026-09

**Authors:** Giulia Faolotto, Anishia Wasberg, Rommel Iheozor-Ejiofor, Essi M. Korhonen, Rosalba Minisini, Tarja Sironen, Ruut Joensuu, Jiaxin Ling, Åke Lundkvist, Olli Vapalahti, Eili Huhtamo, Paolo Ravanini

**Affiliations:** Maggiore della Carità University Hospital, Novara, Italy (G. Faolotto, P. Ravanini); Uppsala University, Uppsala, Sweden (A. Wasberg, J. Ling, Å. Lundkvist); University of Helsinki, Helsinki, Finland (R. Iheozor-Ejiofor, E.M. Korhonen, T. Sironen, R. Joensuu, O. Vapalahti, E. Huhtamo); University of Eastern Piedmont, Novara (R. Minisini); University of Helsinki and Helsinki University Hospital, HUSLAB, Helsinki (O. Vapalahti)

**Keywords:** hantavirus, viruses, zoonoses, arenavirus, Dobrava-Belgrade virus, Puumala virus, Orthohantavirus, lymphocytic choriomeningitis virus, Italy

## Abstract

Rodentborne hantaviruses and lymphocytic choriomeningitis virus (LCMV) circulate in northern Italy but are rarely diagnosed. We tested samples collected from 371 patients during 2004–2018 and detected LCMV IgG in 2.7% of samples and hantavirus IgG in 7.3%. Puumala virus–neutralizing antibodies were confirmed in 10 cases, indicating unrecognized local hantavirus circulation.

In Europe, endemic rodentborne viruses include Old World hantaviruses (Hantaviridae) and lymphocytic choriomeningitis virus (LCMV; Arenaviridae) (*1*). Puumala virus (PUUV), carried by the bank vole (*Clethrionomys glareolus*), is the most widespread hantavirus in Europe. In southeastern Europe and the Balkans, Dobrava-Belgrade virus (DOBV) in yellow-necked mice (*Apodemus flavicollis*) and Saaremaa virus in striped field mice (*A. agrarius*) are also present (*1*). Those mouse species and other potential reservoir species, such as the *Mus musculus* mouse and *Rattus norvegicus* rat, are common in northern Italy, including the regions discussed in this article (*2*). 

DOBV and PUUV cause hemorrhagic fever with renal syndrome (HFRS), and LCMV, transmitted by house mice (*M. musculus*), can cause febrile illness or aseptic meningitis (*1*). Unlike in many neighboring countries, rodentborne infections are not commonly diagnosed in Italy, although evidence of LCMV and DOBV circulation and human exposure exists (*2*–*6*). This study aimed to determine serologic evidence of hantaviruses and LCMV exposure in northwestern Italy, a region where these viruses have not been extensively studied.

## The Study

The study samples (n = 371, average age 59.9 years) were collected during 2004–2018 for other studies in the provinces of Novara, Biella, Vercelli, and Verbano Cusio-Ossola (Figure) and included patients with various chronic diseases, including nephrologic pathologies, alcohol abuse, hepatitis C virus infection, and liver diseases. All patients provided written informed consent, and the study was approved by the local ethics committee. In 2018, we screened patient plasma samples for PUUV, DOBV, and LCMV IgG using immunofluorescence assay (IFA) as previously described (*2*). To further determine hantavirus antibody specificity, we tested neutralizing antibodies in 2024 and 2025 against PUUV and DOBV for samples with IgG titers >1:40 in DOBV or PUUV IgG IFA.

**Figure:**
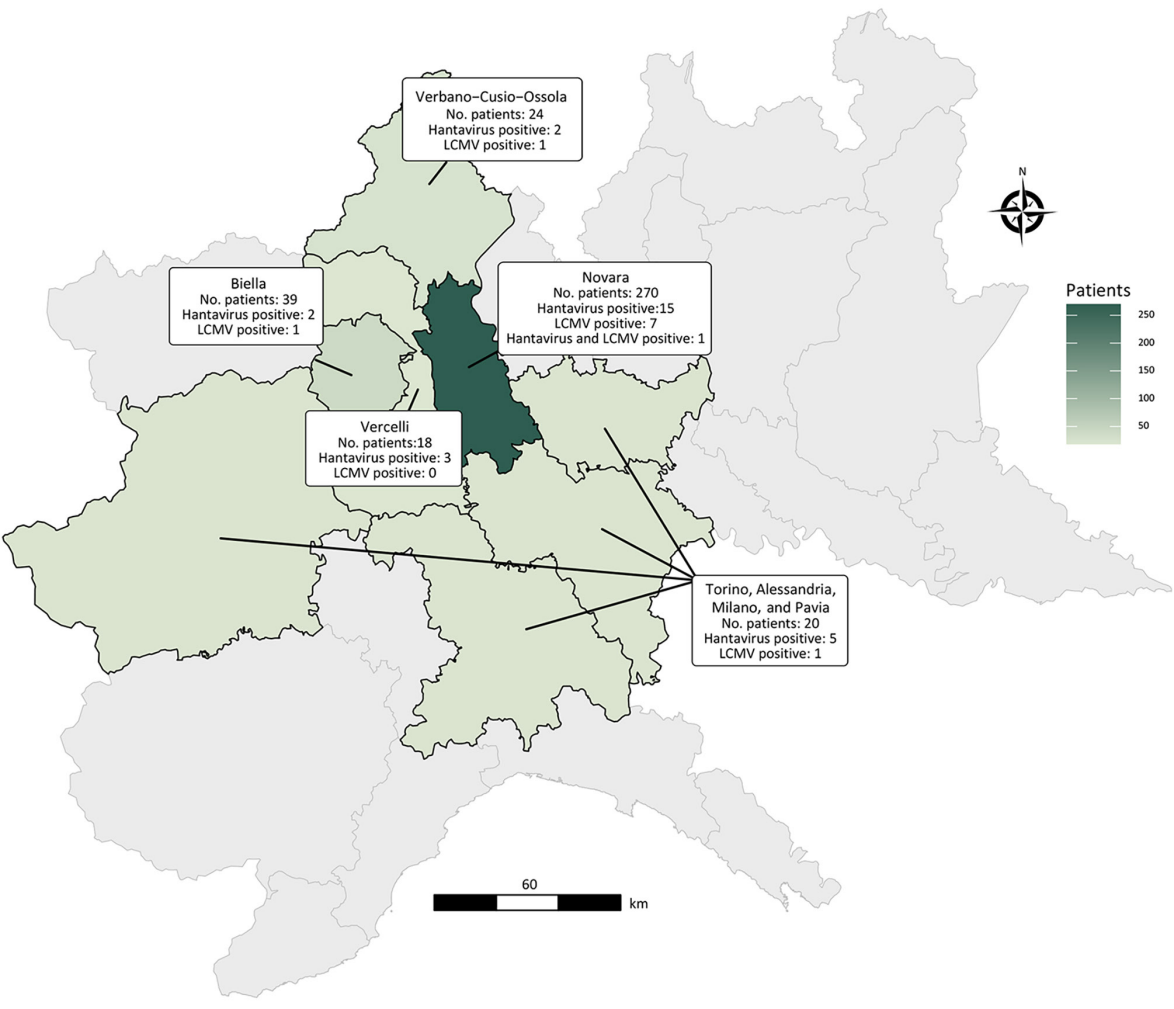
Geographic distribution of study participants and seropositive cases by region in study of serologic evidence of hantavirus exposure in humans, Italy, 2004–2018

We performed focus reduction neutralization tests (FRNTs) in Vero E6 cells according to previously published protocols (*7*,*8*). In brief, we incubated Vero E6 cells with serially diluted plasma samples and hantavirus (50 focus-forming units/well) in 24-well plates. After adsorption, we overlaid cells with MEM-agarose and incubated for 8–10 days, depending on the virus. We detected foci by immunostaining with a hantavirus-specific monoclonal antibody and horseradish peroxidase–conjugated secondary antibody, visualized with AEC (3-amino-9-ethylcarbazole) substrate. We determined neutralizing titers on the basis of the reduction in foci relative to virus-only controls; we used an 80% reduction relative to the negative control as the cutoff for enhanced specificity.

Of 371 patients, 27 (7.3% [95% Wilson CI 5.0%–10.4%]) were positive for hantavirus IgG by IFA (titers 1:20–1:640) (Table 1). We further tested 11 with titers >1:40 by FRNT and detected PUUV-neutralizing activity in 10/11 of the tested samples (titers 1:80 to >1:320). Neutralization of DOBV was generally lower (titers <1:40 to 1:80). Several samples exhibited PUUV-specific neutralization with minimal or absent DOBV-neutralizing activity (Table 2). For LCMV, 10 of 371 patients (2.7% [95% Wilson CI 1.5%–4.9%]) were IgG positive (titers 1:20 to 1:40) (Table 1). Seropositive cases were geographically dispersed (Figure), and antibodies to both viruses were detected only in the region of Novara.

**Table 1:** Antibody screening results determined by immunofluorescence assay in study of serologic evidence of hantavirus exposure in humans, Italy, 2004–2018

Classification/patient group description	Sampling year	No. (%) patients				
		Total	Hantavirus IgG positive			LCMV IgG positive
			PUUV	DOBV	Combined	
Alcohol abuse	2013–2016 (mostly 2013)	28 (7.55)	2 (7.14)	1 (3.57)	3 (10.71)	0
Liver diseases	2004, 2009, 2012 (mostly 2012)	177 (47.71)	5 (2.82)	11 (6.21)	16 (9.04)	4 (1.69)
HCV infections	2018	30 (8.09)	0	0	0	1 (3.33)
Renal diseases	2006, 2007, 2018	110 (29.65)	3 (2.72)	3 (2.72)	6 (5.45)	5 (4.55)
Oral anticoagulant treatment	2007	26 (7)	1 (3.85)	1 (3.85)	2 (7.69)	0
Total		371 (100)	11 (2.96)	16 (4.31)	27 (7.28)	10 (2.70)

*DOBV, Dobrava-Belgrade virus; HCV, hepatitis C virus; LCMV, lymphocytic choriomeningitis virus; PUUV, Puumala virus.

**Table 2:** Hantavirus IFA and FRNT results among participants in study of serologic evidence of hantavirus exposure in humans, Italy, 2004–2018*

Sampling year	Location (Province)	Sample code	IFA results, titer			FRNT assay results, titer	
			PUUV	DOBV		PUUV	DOBV
2004	Abbiategrasso (Milano)	681	<10	40		160	<40
2005	Novara (Novara)	791	80	80		160	80
2011	Arona (Novara)	S09	40	<10		80	40
2012	Galliate (Novara)	S65	40	<10		80<160	40
2012	Novara (Novara)	S42	80	80		160	40
2012	Castelletto sopra Ticino (Novara)	S22	80	40		80	<40
2012	Novara (Novara)	S52	320	640		ND	80<160
2012	Robecchetto con Induno (Milano)	S18	40	<10		80	40<80
2013	Trecate (Novara)	E19	40	10		80	40<80
2015	Piedimulera (Verbania)	E27	80	<10		160<320	40
2018	Galliate (Novara)	N04	40	<10		160	80

*Average age of participants was 58.9 years; participants were 63.6% male and 36.3% female. DOBV, Dobrava-Belgrade virus; FRNT, focus reduction neutralization test; IFA, immunofluorescence assay; ND, not determined; PUUV, Puumala virus.

This study detected antibodies against hantaviruses and arenaviruses in patients from a previously unstudied region of northwestern Italy. Hantavirus infections are routinely reported in neighboring countries but are rarely diagnosed in Italy; yearly cases have been reported in France, Austria, and Slovenia (*9*). LCMV seroprevalence (2.7%) matched previous reports from the Trentino area (*2*). Our hantavirus seropositivity (7.3%) was higher than the 0.2% DOBV IgG reported for forestry workers in Trentino in 2002 (*2*) and above the estimated 1.7% average in the general population in Italy (*10*). Higher seroprevalence (up to 8.8%) has been reported in occupationally rodent-exposed risk groups (*5*).

IFA results demonstrated that plasma samples were positive for PUUV or for DOBV IgG; however, FRNT demonstrated predominantly PUUV-specific neutralizing activity, suggesting PUUV as the likely infecting virus. Neutralization against DOBV was absent or detected at low titers, consistent with serologic cross-reactivity rather than an actual DOBV infection. Only 1 sample (S52) showed reactivity to both PUUV and DOBV antigens by IFA and had a mixed neutralization profile. That finding could indicate exposure to an antigenically related hantavirus or the presence of cross-reactive antibodies. Cross-neutralizing antibody responses among hantaviruses have been documented in both acute and late-convalescent patient samples, potentially complicating serologic interpretation. Although dual exposure to both PUUV and DOBV cannot be excluded, the serologic findings do not enable a definite distinction between these possibilities (*7*,*11*)

The first limitation of our study is that single-time-point sampling precluded infection timing and symptom linkage to seropositivity. Because samples were collected from chronically ill persons, the findings might not fully represent the general population, and data on risk factors, residence, and travel history were unavailable. Residence in a large metropolitan area such as Milan could argue against local transmission; however, patients’ chronic conditions and regular follow-up at the regional university hospital support predominantly local or regional exposure. Furthermore, geographic representation was uneven; most patients resided in the Novara area, potentially biasing the spatial distribution of seropositive cases.

Overall, we found no clear association between serologic findings and patient group, location, or sampling timing. Most hantavirus-seropositive patients were men 36–88 years of age, consistent with reports of higher incidence of hantavirus infection in men and a predominance in the 45–64–year age group (*9*).

The neutralization profile strongly suggested exposure to PUUV or a closely related hantavirus rather than DOBV, previously reported in Italy (*2*,*4*–*6*). The symptoms of PUUV and DOBV infections can vary from asymptomatic to severe and often include fever, headache, abdominal pain, backache, and nausea, occurring in the absence of respiratory symptoms (*1*). Although both viruses can cause HFRS, disease associated with DOBV is more severe (5%–10% fatality rate), whereas PUUV infection is typically milder (<1% fatality rate) (*1*), which might hinder clinical recognition. PUUV infections have been documented in neighboring countries in the Alpine region and central Europe (*12*–*14*) and, given the continuity of forest ecosystems and the distribution of the bank vole, its presence in northern Italy is plausible. Although the bank vole, the natural host of PUUV, typically inhabits forests, our study area was largely semiurban and agricultural with fragmented woodland. How human exposure occurs in such environments warrants further investigation. Of note, a recent PUUV outbreak in coastal Croatia (*15*) highlights the virus’s ability to emerge beyond typical bank vole habitats.

## Conclusions

Our serologic findings in a cohort of chronically ill patients in northern Italy demonstrated exposure to hantaviruses and LCMV. The rate of hantavirus antibody detection was notably high, suggesting local transmission. Together with previous reports from Italy, our findings highlight that rodentborne viruses, particularly hantaviruses, are likely endemic beyond the currently recognized regions. Because of limited data on human infections, hantaviruses remain largely underdiagnosed and neglected in Italy. Although the most recent samples in this study date from 2018, the detection of antibodies across multiple sampling years suggests sustained exposure in this region. Further studies in both rodents and humans are needed to identify endemic hantavirus species and better estimate associated risks and disease burden. Raising clinician awareness of mild forms of HFRS, such as nephropathia epidemica, and ensuring access to appropriate laboratory diagnostics are crucial for timely detection and confirmation of infections. Public health messaging would be essential to minimize exposure to rodents and their excreta, helping to prevent rodentborne diseases across Italy.
